# New York Odontological Society

**Published:** 1894-06

**Authors:** 

**Affiliations:** New York Odontological Society


### Reports of Society Meetings.

### NEW YORK ODONTOLOGICAL SOCIETY.

A REGULAR stated meeting of the New York Odontological Society was held on Tuesday evening, March 20, 1894, at the New York Academy of Medicine, No. 17 West Forty-third Street, New York City, Dr. Brockway presiding.

The Secretary read the minutes of the last meeting, which were approved.

INCIDENTS OF OFFICE PRACTICE AND CASUAL COMMUNICATIONS.

*Dr. S. G. Perry.*—At the last meeting, Dr. Charles Allan spoke of the difficulty of obtaining rubber-dam clamps accurately adjusted to teeth. I thought at the time of some clamps that I had made about two years ago, and which have been in satisfactory use ever since. They are made in such a manner as to be accurately adapted to the shapes of the teeth, but differing a little from those that are generally used in having the bows so small that the rubber dam does not pull upon them, and also in having substance enough in the body of the clamp to give stiffness and firmness, so that they stay where they are placed. They are made in rights and lefts, three different sizes in each kind, and are adaptable to almost any size of upper molar. I never had time to adapt them to the lower teeth, but some day I hope to do that. They were made after patterns which I made with my own hands, and then I had Mr. Drum copy them for me. They are tempered in such a way that they hold very well. Wherever they are placed they will stay. (Since these were shown I have had a set adapted to the lower teeth, made in the same general way.—S. G. P.)

*Dr. S. E. Davenport.*—It having come to my knowledge that Dr. J. N. Davenport, of Northampton, Mass., was experimenting with aluminum for artificial dentures, I requested him to exhibit a plate before this Society, and he has sent the first one he ever made,—a dummy plate,—and with which, as you will notice, he has taken no pains to give the teeth the proper curves. I presume we have all heard more of the cast aluminum plate than we have of the swaged plate; at least, that is true of myself; and while the latter was not absolutely new to me, it was comparatively so, and I thought it might interest the gentlemen to see it. It is swaged of one piece of metal, the band being turned over, as is often the case in gold work, and the plate etched afterwards, so that the teeth may be attached with rubber. The aluminum for these cases is made by the Florence Manufacturing Company, of Florence, Mass., and the manufacturers, who make it for use in the arts almost entirely, claim that the care taken in purifying the metal makes it of better quality for dental use than that which is usually offered by the dental depots. The metal selected for these cases is American guage, No. 26, of which I have a sample here and will pass it around with the plate. There is one thought which comes to me regarding the advantage a swaged aluminum plate has over a cast one, and that is the increased density and specific gravity of the metal, which comes from the necessary force given in swaging, while the cast plate, the metal having been so recently fused, must possess less strength and firmness.

*The President.*—Have you any knowledge of its enduring qualities,—whether it lasts or not?

*Dr. Davenport.*—I can say nothing whatever about that, because it is a comparatively new idea with Dr. Davenport. He is only experimenting with it at present, and the reason he was willing to exhibit the plate before the Society was the hope that some of the gentlemen would be willing to express themselves regarding the usefulness of aluminum in this form. He is very anxious to have comments made, and will be thankful for any information from those who have had experience with aluminum plates.

*Dr. Woodward.*—I will say that some two or three years ago I put two entire pieces in a mouth,—that is, upper pieces,—and one of them kept perfectly bright, as bright as the day on which it was put in; while the other one, made of the same metal, at the same time, of the same quality, by the same person, turned as black as could be, and no amount of polishing from day to day would cause it to keep its color. I saw the other piece to-day, and it was as bright as ever.

*The President.*—Were they swaged or cast?

*Dr. Woodward.*—Cast; they were made at the same time and probably of the same material. Both of them had the peculiarity of shrinking away from the rubber, or rather they would leave the rubber. I thought at first the rubber left the metal, but I think now the metal was destroyed probably by the sulphur in the rubber. The plate that is now worn lasts about six months and then requires a new rubber. The lady, however, values it highly.

*Dr. Sanger.*—I had quite a little experience in that line. I swaged a number of plates and put two of them in the mouth, and I had a peculiar experience with one of them. The patient wore it satisfactorily for about a year, and at the end of that time she returned to me with the air-chamber entirely dissolved out of the plate, not worn through. I say that because of the peculiar appearance of the edges. This being the thinnest point in the plate, in swaging it had given out first. The plate showed signs of dissolving at other points, but the air-chamber was out entirely. It rather shook my faith in swaged aluminum plates, and I abandoned them without giving them a further trial. I put this plate in the mouth of a colored servant of mine, so I could watch it, with that interesting result. I showed that, together with some plates swaged by myself, at a clinic at the New York College of Dentistry, about four or five years ago. There was not much trouble in swaging it. It requires a little care.

*Dr. Littig.*—I have had considerable experience with this class of work. I have always believed the deterioration of the metal was due to some peculiar condition of the secretions of the mouth, caused by the kind of food used, more especially with those who take a large amount of salt. Occasionally we find a certain amount of disintegration of the plate coming up in the form of blisters, and there would be a clayey substance under them. Whether that is due to impurities in the metal or not I cannot say. In some mouths it disintegrates very quickly, and in others the plates wear very well and give good service. I have some that have been worn for a number of years. It is not reliable, however, in this respect. (I am speaking of swaged plates.) In regard to annealing it, by simply putting oil on the plate and then burning the oil off and heating it until it becomes white, that anneals it very nicely.

*Dr. Bogue.*—At our last meeting I was so much obliged to Dr. Perry for his matrices that I thought I would take the liberty of showing one that I have had for a number of years. The manner of using the instrument is to pass the matrix between two teeth, at a point where the matrix is very thin, draw it through until you obtain some pressure, when you will have between the teeth a matrix which is thick at the bottom, towards the gum, and comes to a knife edge at the grinding surface of the teeth to be filled. A bit of thin ribbon steel can be placed between it and the cavity, so if one uses amalgam, the matrix can be drawn down and pushed out again without disturbing the filling, and the steel ribbon removed afterwards.

Another little appliance I wish to speak of. We are often annoyed by the shank of the bur in the engine catching the rubber dam. A tube an inch or two long, that fits over the nozzle of the hand-piece, may be filed away, so as to leave a little finger projecting the same distance as the shank of the bur would project. When this is slipped on the hand-piece, the bur being in place, there is no danger of the shank catching in the rubber dam.

*Dr. Hodson.*—It has been upon my mind for a long time to present to you a very delicate, very simple, but strong, little matrix, which I have used with much comfort for several years, and will, with your permission, present it now.

To premise, I have never felt justified in using the matrix before devising this one, as all others that I have ever seen—excepting Dr. Jack's, and that is thick and requires much space—are practically but straight bands encircling the tooth, and giving convexity but in one direction. One is scarcely better off at the end of using them than with none, as the same straight cut across is left in the former case as the file would make in the latter. In a word, I feel the necessity quite as great for convexity of the stopping from grinding surface to cervix, as from buccal to lingual surface. This I have never seen accomplished by any matrices.

I cut a small piece, sufficient to cover the approximal surface of the tooth, from thinnest ribbon steel, after drawing the temper, then actually stretch, not merely bend, it concave upon a piece of lead by striking with a hammer a small convex tool held upon it; a tiny hole is punched through each side at the extreme edge, in which to insert little pointed instruments when it needs to be moved or withdrawn. This is slid with a curving motion into the space, being helped to its position with, say, a large plugger. If it strikes the cervical edge of the cavity, put a plugger point into the cavity against the edge that catches, and gently pry it away while you push the matrix past it and home. This all takes but three or four minutes to make and apply, though I keep on hand a lot of different sizes and convexities, from which almost any presentation may be fitted.

Once adjusted it fits like a glove, and the stiffness produced by the hammering, combined with the impingement of the convex side against the contiguous tooth, keeps it snugly in place; while, on the other hand, the edges, being free and springy, admit of the production of absolutely perfect filling margins. In some cases—like long teeth—it is prudent to make the matrix narrow, and then slip it upward as the filling progresses. The entire finishing (of course, after very thin burnishers) is accomplished with the thin approximal tapes. So exquisitely do these slips of matrices do their part of the work, that I often build up two contiguous surfaces with no previous wedging, and, while the cervical and other margins are perfect, the two rounded surfaces of the stoppings are so closely adjusted to each other that the floss just catches—as it should—at the convexity. In the case of amalgam stoppings, I usually leave the matrix *in situ* over night, being careful, of course, in its adjustment, that the antagonizing tooth does not hit it.

*Dr. Bogue.*—In continuation of Dr. Hodson's remarks, I would like to call attention to the fact that the matrix which I showed permits the gold to lap over at all sides of the margin of the cavity except the edge next the gum. This surplus may be condensed at the edges with a thin burnisher, so that the gold is more compact than by the ordinary processes of matrix-filling.

*Dr. Hodson.*—The point I wanted to make was that any matrix I have ever seen (Dr. Bogue's included) leaves a straight surface across or between the teeth almost exactly similar to what a separating-file would leave. Where do we get the value of the matrix under such circumstances? To be sure, they make a curve going between the teeth, but never any rotundity from the cervix to the grinding surface. I never could make perfect rotund fillings by any matrix I have ever seen, with the exception, as before mentioned, of Dr. Jack's, and these are thick and require large space.

*Dr. Carr.*—I will cite a case which has greatly interested me, from the fact that it is the only one of the kind that ever came under my observation. A gentleman, about fifty years of age, consulted me eighteen months ago, suffering from what he believed to be chronic rhinitis. Upon careful examination I found a profuse discharge from the left posterior nares, complete anosmia, and deafness, together with considerable pain upon the affected side. I diagnosed empyema of the antrum, and suggested treatment which he considered rather heroic. Consequently, as he was soon going to Europe, he preferred to wait and consult a surgeon while there. He did so, and found my diagnosis confirmed, which decided him to return to me for treatment. After treating him some time without marked improvement, I made an examination of the floor of the antrum, supposing that the septum of bone often found in the floor had been enlarged so as to divide the sinus into two portions. But, to my surprise, I found a large movable body, located at the point corresponding to the posterior buccal root of the second molar, which proved to be an alveolar abscess. The first and second molars had been removed about ten years previously. Evidently the roots of the second molar had perforated the sinus, and when the tooth was extracted the abscess remained attached to the alveolus. Since the removal of the abscess his hearing is restored, the anosmia is gradually disappearing, and the discharge of pus has nearly ceased.

*Dr. Perry.*—How large was the opening?

*Dr. Carr.*—The abscess was removed by enlarging the original opening to about one inch and a quarter in circumference.

*Dr. Perry.*—I would like to say a word that may be of practical value to some one. An eminent surgeon came to me the other day with one of his fingers wrapped up. He said that, while operating, a little poison had got under the skin through a slight scratch. I asked him his remedy, and he said the best one he had found was acetic acid, which he applied after sucking the wound. He considered that better than anything he had ever tried. It penetrated promptly and quickly, and destroyed the poison with more certainty than carbolic acid or iodoform, or anything he knew of. He spoke rather disparagingly of carbolic acid, but he said acetic acid would penetrate promptly, and was almost certain to act on the poison and effect a cure. He told me the ordinary acetic acid fluid was used. He is an eminent man, and this hint from such authority is worth noting.

Dr. C. E. Francis then read the paper of the evening, entitled "Soft Foils and Plastic Stoppings."

(For Dr. Francis's paper, see page 356.)

DISCUSSION.

*Dr. Francis.*—Since our last meeting I have filled several teeth with tin-foil and some with tin shavings. One of the teeth here exhibited is almost wholly filled with tin-foil, but near the upper border gold was added, and the gold worked as nicely with the tin as if they had been the same metal. I have observed on a number of occasions that manufacturers of appliances, instruments, materials, etc., have been invited to attend meetings of our Society, in order that they might be questioned. I took the liberty of inviting Mr. Kearsing, of Brooklyn, to come here, and if any one wishes to interrogate him, I presume he will be glad to answer questions. Since Professor Darby introduced his new form of tin, I have probably used more tin than in several years previously. The more I use it, the better I like it. There are a certain class of cavities, especially large ones, in which it serves an excellent purpose. I feel safe in using it. I would say, in regard to the fillings in the teeth that are being passed around, the one with a wire attached is filled with Professor Darby's tin shavings, and the others with Mr. Kearsing's tin-foil.

*Dr. Littig.*—Does tin-foil retain its cohesive qualities for any length of time?

*Mr. Kearsing.*—I think it will. There is nothing but the tin there. I think it will hold until some acid gets hold of it.

*Dr. Jarvie.*—Will it oxidize from the atmosphere, and so have its cohesive properties destroyed?

*Mr. Kearsing.*—No, sir.

*Dr. Jarvie.*—How long have you been making this cohesive tin-foil?

*Mr. Kearsing.*—A great many years.

*Dr. Hodson.*—Is there any way of putting the cohesion back again if it should be lost?

*Mr. Kearsing.*—Not that I know of.

*Dr. Lord.*—I suppose that Dr. Francis does not mean that the tin is cohesive as gold is cohesive, but it is made cohesive by properly manipulating it,—that is, we may call it cohesion or mechanical welding. It will not stick without pressure, and without pressure by points. Cohesive gold will cohere or adhere without any pressure.

*The President.*—Does Dr. Francis discover any perceptible difference in the way of working cohesive tin from cohesive gold?

*Dr. Francis.*—It works much more easily, and does not offer any resistance.

*Dr. Ives.*—When you add a fresh piece to your filling, does it cohere? You know how gold does.

*Dr. Francis.*—It seems to cling very easily.

*Dr. Jarvie.*—I have not seen the tin-foil, but the tin shavings will.

*Dr. Hodson.*—A leaden bullet, cut in two and the fresh surfaces rubbed together, will cohere and stick together, so that the fresh surface of the pure metal—gold or tin—would have the same cohesion as the lead.

*Dr. Francis.*—This foil works much better than the old tin-foil.

*Dr. Lord.*—I suppose Dr. Francis does not mean that tin is cohesive in the sense that cohesive gold is cohesive. It undoubtedly has cohesive properties, but not such as the gold has. I do not in the least propose to depreciate this foil, but to call it cohesive tin would not be just the term to use. As we lay one sheet upon another, or fold them together, there is no cohesion, yet in the operation of packing and condensing with proper instruments we can make the filling solid, not unlike molten metal. I should say that this ought to be termed mechanical cohesion.

I have taken the liberty of saying to Mr. Williams, from time to time, that the tin-foil that he made twenty years ago was better than that which he has made since or makes now, but he thinks I am mistaken.

It will be seen that this tin is just as bright as when it was first made, and it works as well. I think it is tougher than Mr. Kearsing's, though not so soft, which is a more excellent quality.

*Dr. Nash.*—Is it the same thickness?

*Dr. Lord.*—Yes, about the same; perhaps a little thicker, and that would help in its toughness.

*Dr. Nash.*—Are the numbers the same?

*Dr. Lord.*—Mr. Williams's is No. 4; Mr. Kearsing's is not numbered.

I have used gold and tin in combination and with great satisfaction. I fold the tin inside the gold in the proportion of about fifteen parts gold to one of tin. In this proportion there will be no discoloration, nor will the tin oxidize nor wash away, and in a short time there will be an amalgamation of the two metals.

*Dr. Perry.*—It seems to me, if I understand this matter, that it is the clean surfaces that make the greater cohesion of the shavings that were cut by Dr. Darby. I was not surprised when I saw the result; it might have been expected. How long that cohesion will stay remains to be seen. I have an idea that it will be lost after exposure to the atmosphere. So with this tin-foil: there will gather upon it gases from the atmosphere which will spoil the cohesion.

*Dr. Lord.*—I have a few thoughts upon the subject, which I would like to present.

(For Dr. Lord's notes, see page 363.)

*Dr. Francis.*—I would much like an expression of opinion as regards the solidity of the fillings in the teeth that have been exhibited this evening. Solid filling only would take a lustre.

*Dr Bogue.*—There was one feature that Dr. Watkins, of Montclair, brought up the other evening at the District Society that I notice has only been touched upon this evening. It is one of so much importance that I want to bring it up again. He stated that the idea of putting a little tin-foil at the cervical margin of approximal cavities and then covering with gold was extremely bad, because wherever you put a little tin and much gold the tin wasted. If you fill a cavity with tin and gold, it should be little gold and much tin. I think Dr. Lord brought out that idea once before, but in a different way. Wherever a little tin bed has been made for the gold to go in, I think it has invariably wasted and left a cavity underneath. I think Dr. Lord is right in saying that tin has been largely abandoned because it was not gold. It is much more easily worked than gold, and it has been a great favorite with me from the time I first started in practice; it has been used in children's teeth especially, and with the utmost pleasure and safety.

Now, a word in regard to Mr. Kearsing's remark that the tin would retain its cohesiveness. I think only time can settle that question, and unless we follow Dr. Lord's example and shut up the tin so its surface cannot oxidize, I see no way to be certain that the cohesive properties will remain.

Wherever pure metal comes in absolute contact with the same pure metal, there will be cohesion, whether it be gold, tin, or lead. Therefore cohesive fillings can be done with tin, and it has been for many years, although rarely. It seems to me the question of what material we use to save teeth should be one of minor importance. The question ought to be that of saving teeth, and the material that we use at one time or another is a question which we should consider after our conditions have been fully discovered.

*Dr. Jarvie.*—If there is one thing I like to do more than another, it is to please Dr. Lord, and so I will take exception on one point as to what he considers the desirable qualities of tin-foil. But before going to that point, let me say that ever since I commenced to practise dentistry I have used tin-foil in certain classes of cavities. I think it is by far the most valuable filling-material we have for cavities in the grinding surfaces of the teeth of children, whether they be temporary or permanent teeth. I do not like the tin-foil as thick as that shown by Dr. Lord, which was made by Mr. Williams several years ago. Tin-foil beaten out thinner than that is, to my mind, preferable; and the reason is that it requires less force to adapt it perfectly to the walls of the cavity and to condense it, and that is a very valuable consideration when we are working upon children's teeth. I do not think that the toughness, as expressed by Dr. Lord, makes very much difference, as long as we get it thoroughly condensed in the cavity. If we get a solid mass of the block tin in the cavity, it does not make much difference whether it is tough or not in the sheet. To my mind No. 4 is not nearly so good for the purpose for which we want it as when it is beaten out thinner. A number of years ago, Mr. Kearsing got out a lot of tin-foil that was not any thicker than No. 3. I purchased from him a number of books at that time, and I am using from that lot to-day. I still have several books that I have not used, and I value it very highly. I suppose I can get more like it, but if I could not, I should be very careful how I used that which I have. It is as precious to me for certain purposes as any gold which I possess.

*Dr Howe.*—I am very glad that this subject of the use of tin has been brought up for discussion, and that expressions of opinion in its favor have been as decided as they are. I think it is one of the best materials we have, but I am afraid it has been very much neglected by many practitioners. I have used tin-foil quite steadily and regularly during all the years of my practice. It seems to me that the possibilities of this new tin-foil that has been used by Dr. Francis so successfully must be greater than anything I have used, for the fillings that Dr. Francis has shown in these teeth to-night are contoured to a considerable extent and are finished very handsomely, and show by the finish a great deal of density. I would like to have Dr. Francis state to us what kind of instruments he used to pack with,—whether they were serrated or not, and, if so, whether they were deeply serrated, and whether he used altogether hand-pressure or whether he used mallet force to some extent.

*Dr. Francis.*—In reply to Dr. Howe's inquiry, I would say I used simply the ordinary pluggers with small serrations, the same as I use for gold-foil. Some of these fillings were in great part packed with hand-pressure. I tried the hand-mallet and the automatic mallet, with about the same effect. The foil works very easily indeed, and seems to pack like magic, readily and beautifully. Two of the teeth I held in my fingers, but the bicuspids were held screwed in a hand-vice. One partly filled with gold is three-quarters tin. I used non-cohesive foil. The first few layers I did not anneal, but the others were annealed.

*Dr. Bogue.*—Were those made with broad points?

*Dr. Francis.*—No; the serrations were very light. The greater part of the filling in each cavity was put in by hand-pressure. One, as I have stated, is filled with Professor Darby's shavings. The tin-foil is more convenient to introduce in the cavities than the shavings.

*Dr. Hodson.*—In respect to the building up of tin-foil (I believe the process to be chiefly one of interdigitation), I am quite sure that I have at home the first tooth I ever filled, twenty-five years since, and that is built up fully a quarter of the crown's size with tin-foil. I think there is nothing specially new about building up tin-foil fillings. I have never used it very much in my practice because my impression has been that its employment was based chiefly upon its non-conducting properties and the assumed facility of its insertion. Inasmuch as it would take me nearly as long to place a tin stopping as one of gold,—granting, of course, equal care with both, —and as I could get equally good results, or better, in respect of non-conduction by treating the surfaces of the cavity walls, I have never felt that I would gain in these respects by using tin. Moreover, my observation has taught me that it wears away very much more than the gold would do in similar exposures, and that, I have felt, made another element of weakness in tin. While in certain circumstances of life, or in certain circumstances of teeth, there is a use for it, I have never in my own practice cared much for it.

*Dr. Ives.*—I think there is a little mistake about the use of this term cohesive. Dr. Francis, in his paper, leads us to believe he has used that term just as we use it in regard to gold. If you take two sheets of cohesive foil, place them together, and apply the slightest pressure, you can hardly separate them. Now, we do not get that effect with tin. Some years ago I built out a lateral incisor with tin-foil; it was done with sharp points, purely mechanical cohesion forcing them together. Does Dr. Francis claim that if he arrives at a certain point in his filling, and adding another piece of tin, he can, with a gentle pressure, cause it to cohere as with gold?

*Dr. Francis.*—You can with the shavings.

*Dr. Ives.*—If not, the tin-foil is not cohesive in that sense of the word. I believe in mechanical cohesion, but I do not think the tin-foil is cohesive in the sense that gold is.

*Dr. Francis.*—I can take a ten-cent piece and build a cone of tin-foil upon it, and it will be hard and solid. If not cohesion, what, then, is the force that holds the particles together? Possibly I do not understand the term "cohesion" as others view it. I supposed cohesion meant the union of particles of metal or other substances of the same nature when brought together. You can place this new foil one layer on another and unite the layers.

*Dr. Ives.*—Can you do it with a smooth-face instrument?

*Dr. Francis.*—The instruments I use are very lightly serrated. I have not tried smooth instruments.

*Dr. Hodson.*—Might I suggest that in the pure metals the cohesion is produced by the absolute expulsion of the intervening layer of air, and so the molecules of metal are brought into absolute contact. If one lays the clean cut surfaces of two soft metals together, as I suggested a few minutes ago with the bullet, and rubs them together, perfect cohesion is obtained, but the two parts of the bullet must be rubbed together in order to expel the layer of air. To merely lay them together will not make them cohere, and in that sense they are not cohesive, like sticky gold-foil. Similarly, if by any means we could get two layers of tin-foil in the cavity perfectly smooth and clean (the hammered surface is not clean in the sense that the cut one is), and could rub one upon the other, we could probably get the same cohesion as in the leaden bullet.

Adjourned.

JOHN I. HART, D.D.S.,
*Editor New York Odontological Society.*

